# Asthma and Rhinitis Are Associated with Less Objectively-Measured Moderate and Vigorous Physical Activity, but Similar Sport Participation, in Adolescent German Boys: GINIplus and LISAplus Cohorts

**DOI:** 10.1371/journal.pone.0161461

**Published:** 2016-08-25

**Authors:** Maia P. Smith, Dietrich Berdel, Carl-Peter Bauer, Sibylle Koletzko, Dennis Nowak, Joachim Heinrich, Holger Schulz

**Affiliations:** 1 Institute of Epidemiology I, Helmholtz Zentrum München – German Research Center for Environmental Health, Neuherberg/Munich, Germany; 2 Research Institute, Department of Pediatrics, Marien-Hospital Wesel, Wesel, Germany; 3 Comprehensive Pneumology Center Munich (CPC-M), Member of the German Center for Lung Research, Munich, Germany; 4 Institute and Outpatient Clinic for Occupational, Social and Environmental Medicine, Ludwig-Maximilians-University, Munich, Germany; 5 Dr. von Hauner Children’s Hospital, Ludwig-Maximilians University, Munich, Germany; 6 Department of Pediatrics, Technical University of Munich, Munich, Germany; University of Oslo, NORWAY

## Abstract

**Introduction:**

Physical activity (PA) protects against most noncommunicable diseases and has been associated with decreased risk of allergic phenotype, which is increasing worldwide. However, the association is not always present; furthermore it is not clear whether it is strongest for asthma, rhinitis, symptoms of these, or atopic sensitization; which sex is most affected; or whether it can be explained by either avoidance of sport or exacerbation of symptoms by exercise. Interventions are thus difficult to target.

**Methods:**

PA was measured by one-week accelerometry in 1137 Germans (mean age 15.6 years, 47% boys) from the GINIplus and LISAplus birth cohorts, and modeled as a correlate of allergic symptoms, sensitization, or reported doctor-diagnosed asthma or rhinitis.

**Results:**

8.3% of children had asthma, of the remainder 7.9% had rhinitis, and of the remainder 32% were sensitized to aero-allergens (atopic). 52% were lung-healthy controls. Lung-healthy boys and girls averaged 46.4 min and 37.8 min moderate-to-vigorous PA per day, of which 14.6 and 11.4 min was vigorous. PA in allergic girls was not altered, but boys with asthma got 13% less moderate and 29% less vigorous PA, and those with rhinitis with 13% less moderate PA, than lung-healthy boys. Both sexes participated comparably in sport (70 to 84%). Adolescents with wheezing (up to 68%, in asthma) and/or nose/eye symptoms (up to 88%, in rhinitis) were no less active.

**Conclusions:**

We found that asthma and rhinitis, but not atopy, were independently associated with low PA in boys, but not in girls. These results indicate that allergic boys remain a high-risk group for physical inactivity even if they participate comparably in sport. Research into the link between PA and allergy should consider population-specific and sex-specific effects, and clinicians, parents, and designers of PA interventions should specifically address PA in allergic boys to ensure full participation.

## Introduction

Asthma, allergic rhinitis, and atopic sensitization (collectively “allergic phenotype”) are associated with significant medical and social morbidity in the developed world[1]and their increasing prevalence in the last decades has yet to be explained. Several mechanisms have been proposed, [1] but many studies have linked allergic conditions with insufficient physical activity (PA) [2–4]. Children with asthma, particularly boys [5, 6], often get less PA than their peers [2, 5, 6] despite the fact that an “exercise prescription” is recommended for asthma management [3] and normal PA participation is a stated goal of asthma therapy. [7]

Discussed drivers of the relationship between allergy and low PA include inadequately controlled asthma [8] or rhinitis[5] with exacerbation of allergic symptoms by PA; exercise-induced bronchoconstriction (EIB) in individuals with heightened airway sensitivity [9] such as occurs with allergic phenotype; low sport participation among children with asthma[2, 10]; and confounding by environmental and socioeconomic factors associated with both PA and allergy, such as overweight, ethnicity or environmental exposures. [6, 11, 12] As a result children with asthma or rhinitis may deliberately avoid active pursuits, be discouraged from sport by their parents or caregivers [2, 10] and/or be unable to participate in vigorous PA (VPA.) [13] Conversely, some evidence suggests that physical fitness may protect against future development of asthma. [14, 15] However, benefits of PA are proven: physical inactivity in any group is a health risk that should be addressed, whether or not these benefits include prevention of allergy.

The association with allergy, while suggestive, is not conclusive. Some studies fail to find associations between allergy and PA in one sex only [5, 16] [17] and others find no association at all [2]. Protocols are not standardized among studies [2] so different approaches to assessing PA and defining allergies may account for some heterogeneity[2] as may issues of sample population and confounding. Allergy may be quantified as any of the several allergic diagnoses, which may not be standardized [18, 19] or confirmed by physician; or it may be based on symptomsreported by parent or child. [2, 13] Likewise, PA may be reported by the child or their parent [20, 21] or measured objectively by accelerometry,[13] and may be quantified as sport participation or as minutes spent in moderate, vigorous or moderate-to-vigorous activity (MPA, VPA, MVPA); studies relying on subjectively-reported PA data tend to assume that most MPA and VPA take place during well-defined sporting activities [2, 10] which is not always the case [22, 23] [24] and thus may overestimate the importance of sport and active lifestyle as a correlate of allergy. For all these reasons, research is difficult to aggregate and may be considered as inconclusive.

In this study we aim to assess relationships between allergic conditions, allergic symptoms, and PA in a large population-based sample of German adolescents. We compare PA levels in adolescents with reported physician-diagnosed asthma and rhinitis, and IgE-determined aero-allergen sensitization, to those in a group of lung healthy controls. PA was measured throughout a representative week by accelerometry, making it possible to separately establish relationships for different indicators of PA level, particularly vigorous activity. We also examine possible mediators of the relationships we find, including sport participation as an indicator of active lifestyle, and activity limitation by allergic symptoms.

## Methods

### Population Characteristics

We combined data from interviews, physical examinations, and accelerometry from two cohorts of German Caucasians: GINIPlus and LISAPlus, born between 1995 and 1999. Further details on study designs of GINIplus [25, 26] and LISAplus [27] are published elsewhere. Both studies were approved by the respective local Ethics Committees (Bavarian General Medical Council, Medical Council of North-Rhine-Westphalia) and by written consent from participating families.

GINIPlus (German Infant Nutritional Intervention PLUS environmental and genetic influences on allergy development) was initiated to investigate allergy development. Of 5991 infants recruited at birth, 2252 had a family history of atopy and thus were given hydrolysed baby formulas. The remainder was given no formula. At age 15, 3199 adolescents were recontacted and approached for accelerometry. 1890 subjects (59%) consented to accelerometry, of whom 1290 (40%) successfully completed and 1054 (33%) passed quality control. Ultimately 847 of these (26%) were included in the present study. For further details on recruitment, formulas, and followup see von Berg et al. (2015) [28]and Smith et al. (2016) [29]; for details on accelerometry response see Smith et al. (2016.) [23]

LISAPlus (Lifestyle-Immune-System-Allergy; Influence of lifestyle factors on the development of the immune system and allergies plus the influence of traffic emissions and genetics) is a population-based cohort of 3097 unselected infants[30] from the cities of Munich, Wesel, Bad Honnef and Leipzig. 1534 subjects were followed up at age 15, of which 1107 (64%) were from Munich or Wesel and thus approached for accelerometry. 655 subjects (59%) consented to accelerometry, of whom 435 (39%) successfully completed and 357 (32%) passed quality control. Ultimately 290 (24%) were included in the current study. [23]

For a flowchart on accelerometry recruitment, response, and completion see Smith et al. (2016.) [23] Of 1411 subjects who completed accelerometry, [23] 1137 (83%) had complete data and were included in the current paper.

### Measurements of PA

Accelerometry was combined with an activity diary to document activity domains and weartime. Detailed descriptions of accelerometer protocol, quality control, and data cleaning are given elsewhere. [31, 32] [23] Accelerometers (ActiGraph GT3X, Pensacola, Florida) were worn at the hip. Sampling rate was 30 Hz; accelerations were stored at 1 Hz and converted into activity levels in one-minute epochs using the algorithm for children from Freedson et al, 2005.[33] Diaried weartime was validated against that indicated by the device according to the algorithm of Troiano et al. (2007) [34], using SAS programs from NHANES[31]. Only data from waking time during diaried and validated monitor wear were used. For more details on accelerometry protocol see Smith et al, [23].

Activity diaries contained lines for time of getting up and going to bed, participation in sport, time and reason of removing the monitor, and other activities in a standardized diary, particularly leisure time sport duration and type. Valid days had at least 10 hours of valid recording, or 7 if the subject was awake for between 7 and 10 hours. Valid subjects provided at least 3 valid weekdays, and one valid weekend day. To profile daily activity, we considered average daily minutes of moderate and vigorous PA (MPA, VPA), and sport participation.

### Sociodemographic and Anthropometric Confounders

All multivariable models were corrected for correlates of PA not of primary interest. These were age, height, study center (Munich or Wesel), season of accelerometry, and nutritional intervention. For details see S1 File. Initial analyses suggested sex-specific results, so all presented models are stratified by sex.

### Allergic Respiratory Conditions

Allergic respiratory conditions were asthma, allergic rhinitis and atopic sensitization to aero-allergens, defined as follows:

- **Asthma:** As in Mölter et al. (2015) [18] and Jarvis et al (2012)[35] current asthma at 15 years was defined as having at least two of the following: doctor diagnosis of asthma ever between age 3–15, current wheezing at 15 years of age, and asthma medication at 15 years of age.
- **Allergic rhinitis:** Allergic rhinitis was defined as a doctor diagnosis of either allergic rhinitis or hayfever at any time in the past year. Adolescents with asthma or asthma medication were treated in the models as asthma and excluded from the rhinitis group, even if they also had rhinitis.
- **Aero-allergen sensitization (atopy):** Atopic sensitization was defined as any sensitization to aero-allergens compared with none, defined as at least one RAST positive (IgE ≥0.35 kU/l) for the following airborne allergens: birch, mugwort, ambrosia, grass, rye, dogs, cats, dust mites (*Dermatophagoides pteronyssinus*) and indoor mold (*Cladosporium herbarum*). Models of atopy excluded children with current rhinitis or asthma, or current medication for rhinitis or asthma.
- **Lung-healthy (control):** Lung-health in this population was defined as: since the age of 3 years no asthma, and in the past year no asthma symptoms (wheezing), no rhinitis, no rhinitis symptoms (nose/eye symptoms; see below), no asthma or rhinitis medications, no positive aero-allergen RAST, or positive bronchodilator response (see Supplement and Miller et al.(2005) [36]for more detail on testing)

#### No child in the study had cystic fibrosis

For further details on definitions see S1 File.

### Allergic Respiratory Symptoms

- **Wheezing:** Self-reported wheezing, whistling or chest tightness in the past year was included as an indicator of incompletely diagnosed or treated respiratory disease, particularly asthma, which may discourage vigorous PA.
- **Nose and eye symptoms:** Self-reported current nose and eye symptoms (runny nose, itchy eyes) in the past 12 months in the absence of a cold were included as an indicator of possible untreated or incompletely treated rhinitis, which may in turn discourage PA.

### Statistical Methods

All calculations were done using SAS 9.2 or 9.3 (Cary, NC.)

Populations (Tables 1 and 2) were compared using nonparametric tests: Kruskal-Wallis for multilevel categorical variables, Wilcoxon’s two-tailed rank-sum test for all others. To model corrected relationships, sex-stratified generalized linear models were used to model PA outcomes as statistical functions of respiratory diseases and conditions, corrected for age, height, study center, nutritional intervention, season of accelerometry (categorical) and parental education.

**Table 1:** Population and Selection.

Trait	Whole 15-year followup Munich and Wesel Birth cohorts GINIplus[28] and LISAplus.[27]		Study population		P for difference if <0.10	
	Boys	Girls	Boys	Girls	Boys	Girls
N	4306		1137		--	
Male (N, %)	2198, 51		538, 47		0.003	
Birthdate (mean)	21 May 1997	5 May 1997	6 June 1997	3 May 1997	--	--
Birthdate (min—max)	16 Sep 1995–31 Jan 1999	11 Sep 1995–31 Jan 1999	25 Sep 1995–22 Jan 1999	12 Sep 1995–31 Jan 1999	--	--
Height, cm; mean (SD)	176 (7.5)	167 (6.3)	177 (7.4)	167 (6.2)	--	--
BMI, kg/m^2^; mean (SD)	20.8 (3.4)	21.0 (3.1)	20.6 (3.0)	21.0 (3.0)	--	--
Nutritional intervention^1^, any vs. none (%)	26	25	28	28	<0.0001	<0.0001
BMI category^2^ (%); p for global null					--	--
Underweight	7.8	6.6	8.8	6.5	*	*
Normal	80	84	80	85	*	*
Overweight	8.0	6.0	8.6	5.4	*	*
Obese	4.1	3.8	2.7	3.5	*	*
From Munich (%)	59	59	64	59	0.006	--
Parents highly educated (%)	65	68	70	72	0.01	0.06
**Allergy group (N, % of those with data)**; p for global null, among those with data					<0.0001	<0.0001
Asthma^3^	139, 11	89, 7.7	53, 9.9	41, 6.8	*	*
Rhinitis but no asthma^4^	169, 14	134, 12	50, 9.3	40, 6.7	*	*
Aero-sensitized but no rhinitis or asthma^5^	410, 34	292, 25	197, 37	166, 28	*	*
Strict lung-healthy (control)^6^	501, 41	646, 56	238, 44	352, 59	*	*
Missing data (N)	979	947	0	0	*	*
**In past year** (%)**:**						
Wheezing	6.1	6.8	6.2	6.7	--	--
Asthma medication	5.6	3.8	9.3	5.4	0.00002	0.01
Rhinitis medication	11.7	9.7	9.9	4.9	--	<0.0001
Eye/nose symptoms	19	18	19	16	--	0.03
Positive bronchodilator^7^, where performed	5.0	2.8	3.0	0.98	0.01	0.0009

Study population compared to full 15-year followup

^1)^ Nutritional intervention was used in the first four months of life in the intervention arm of GINIplus. Formulas were partially and extensively hydrolysed whey, extensively hydrolysed casein, or cow’s milk. No intervention was used in the observation arm of GINIplus or in LISAplus.

^2)^ BMI categories from 10^th^, 90^th^, and 97^th^ percentiles for that age and sex in a German reference population (Kromeyer-Hauschield, 2001).

^3)^ Asthma: As in Jarvis et al (2012)[35]: at age 15 the subject reported at least 2 of the following traits: asthma medication or wheezing in past 12 months, doctor diagnosis of asthma at any time since age 3.

^4)^ Allergic rhinitis: Current rhinitis or hay fever at age 15, but no asthma or asthma medicine

^5)^ Atopy: No asthma, no allergic rhinitis, but one or more positive RAST (IgE ≥0.35) for aero-allergens (birch, mugwort, ambrosia, grass, rye, dogs, cats, dust mites *(Dermatophagoides pteronyssinus)* and indoor mold (*Cladosporium herbarum)*

^6)^ Lung-healthy: No asthma ever; no current allergic rhinitis; no wheezing or nose/eye symptoms in past year; no current drugs for asthma or rhinitis; no RAST over 0.35; no positive bronchodilator response.

^7)^ Bronchodilator response is an indicator of current airway hyperresponsiveness, such as may be caused by untreated asthma or recent infection. Testing was performed and defined as in Miller et al (2005) and Flexeder et al (2015)[38]

All measures except bronchodilator response are given only as percentage of subjects with data. P-values from Wilcoxon’s two-tailed rank-sum test for binary variables, Kruskal-Wallis for categorical. —if p>0.10, * if pairwise test not performed (see test for global null in top row.)

GINIplus cohort profiled in von Berg et al, 2015[28]; LISAplus cohort profiled in Chen et al, 2007.[27]

**Table 2:** Population and Selection. Comparison between lung-healthy controls and children with respiratory allergies.

Trait	Boys				Girls				P-value (Group) if p ≤ 0.10, for pairwise difference from controls	
	Lung-healthy^4^	Atopy^3^	Rhinitis^2^	Asthma^1^	Lung-healthy^4^	Atopy^3^	Rhinitis^2^	Asthma^1^	Boys	Girls
N	238	197	50	53	352	166	40	41	--	--
Age, years; mean (SD)	15.6 (0.5)	15.5 (0.5)	15.6 (0.5)	15.6 (0.5)	15.6 (0.5)	15.6 (0.5)	15.6 (0.5)	15.7 (0.6)	--	--
Height, cm; mean (SD)	177 (7.4)	177 (7.3)	174 (7.7)	176 (6.9)	167 (6.0)	167 (6.6)	176 (6.9)	168 (5.8)	0.08 (1);0.006 (2)	0.10 (1)
BMI, kg/m^2^; mean (SD)	20.7 (3.2)	20.4 (2.8)	20.4 (3.2)	21.0 (3.0)	20.9 (3.0)	21.1 (2.8)	21.0 (3.0)	20.8 (3.0)	--	--
Munich, %	61	65	76	62	57	64	55	54	0.04 (2)	--
Parental education, %	72	69	71	67	71	73	68	78	--	--
**In past year, %:**										
Wheezing	0	2.0	12.0	46	0	4.9	10	68	*	*
Asthma medication	0	0	0	94	0	0	0	78	*	*
Rhinitis medication	0	0	66	38	0	0	61	15	*	*
Eye/nose symptoms	0	18	88	47	0	27	84	40	*	*
Bronchodilator response^5^; P for global null									*	*
Negative	82	79	74	74	85	87	80	73	*	*
Positive	0	2.0	6.0	11	0	1.2	2.5	4.9	*	*
Not performed	18	19	20	15	15	12	18	22	*	*
**Physical activity, min** Mean (Geometric mean) 5^th^, 95^th^ percentile										
Moderate	31.8 (30.2) 14, 54	31.1 (29.8) 14, 53	28.7 (27.1) 13, 54	29.6 (26.8) 9.3, 56	26.4 (24.7) 10, 49	25.4 (23.6) 8.8, 45	23.2 (23.0) 13, 37	29.9 (26.2) 12, 50	--	--
Vigorous	14.6 (11.5) 2.1, 39	13.6 (11.4) 2.0, 33	12.9 (11.3) 1.9, 30	11.2 (8.6) 1.0, 31	11.4 (8.8) 0.9, 34	10.7 (8.5) 1.0, 30	10.5 (8.8) 1.4, 26	12.8 (10.2) 1.3, 25	0.06 (1)	--
MVPA	46.4 (42.4) 19, 89	44.7 (41.7) 18, 79	41.6 (38.8) 16, 73	40.7 (35.4) 11, 89	37.8 (34.1) 13, 70	36.1 (32.7) 13, 69	33.8 (31.8) 16, 61	42.7 (36.3) 14, 71	0.05 (1)	--
Any reported sport, %	71	77	80	70	70	84	70	83	--	0.09 (1); 0.08 (3)

^1)^ Asthma: As in Jarvis et al (2012): at age 15 the subject reported at least 2 of the following traits: asthma medication or wheezing in past 12 months, doctor diagnosis of asthma at any time since age 3.

^2)^ Allergic rhinitis: Current rhinitis or hay fever at age 15, but no asthma or asthma medicine

^3)^ Atopy: No asthma, no allergic rhinitis, but one or more positive RAST (IgE ≥0.35) for aero-allergens (birch, mugwort, ambrosia, grass, rye, dogs, cats, dust mites *(Dermatophagoides pteronyssinus)* and indoor mold (*Cladosporium herbarum)*

^4)^ Lung-healthy: No asthma ever; no current rhinitis; no wheezing or nose/eye symptoms in past year; no current drugs for asthma or rhinitis; no RAST over 0.35 or positive bronchodilator response.

^5)^ Bronchodilator response is an indicator of current airway hyperresponsiveness, such as may be caused by untreated asthma or recent infection. Testing was performed and defined as in Miller et al (2005) and Flexeder et al (2015)[38] Reasons for not performing the test included subject refusal and medical contraindications; refusal was the most common.

P-values from Wilcoxon’s two-tailed rank-sum test for binary variables, Kruskal-Wallis for categorical.

–if p>0.10, * if pairwise test not performed (see test for global null in top row, or characteristic was used to define groups.)

Each PA measure (MPA, VPA, and sport participation) was modeled as function of all confounders not of primary interest, as well as one respiratory condition or diagnosis at a time. Odds of any sport participation during accelerometry were modelled logistically and are presented as odds ratios. Daily minutes MPA and VPA were log-transformed for normality before modeling, and inspection of histograms and q-q plots confirmed normality. Results are presented as percent difference from children without the symptom (in models of symptoms) or as percent difference from apparently lung-healthy controls (in models of asthma, rhinitis, and atopy.)

### Inclusion Criteria

To separate the effects of the different allergic diagnoses, these conditions were modeled separately[37] similar to the comparisons made in Mitchell et al, 2013.[4] Children who confirmed asthma (n = 94) were one group; of the remainder, those who confirmed allergic rhinitis were another group (n = 90); of the remainder, those with positive aero-allergen RAST were the third group (n = 363). Children with each condition were compared only to lung-healthy controls (detailed above; n = 590.) However, all subjects were included in the models of symptoms.

Children who did not confirm any diagnosis, but who also did not fit our criteria for lung-health, were excluded from the analysis. For example, this included children who did not confirm asthma or allergic rhinitis and who did not have a positive RAST, but had a positive bronchodilator response (n = 41) and/or asthma in childhood (n = 49) medications for asthma (n = 2) or rhinitis (n = 5), nose/eye symptoms (n = 51) or wheezing (n = 25.) Ultimately of 1411 subjects who completed accelerometry, 1137 (81%) were included in the paper.

## Results

We combined data from the 15-year followup of two large German birth cohorts, GINIplus [26, 28] and LISAplus, [27, 30] conducted in the suburban region of Wesel (north-west Germany) and the urban area of Munich, primarily aimed at the benefit of nutritional intervention for prevention of allergies (GINIplus) and the influence of lifestyle factors on the development of the immune system (LISAplus). Physical activity levels in this cohort have been previously profiled, as has the allocation of activity by domain. [23]

In the study population of 1137 subjects (47% male), asthma, rhinitis, and atopic sensitization were all more prevalent in boys than girls (Table 1): 9.9 and 6.8% of boys and girls had asthma; of the remainder 9.3 and 6.7% had rhinitis; of the remainder 37 and 28% were atopic (here defined as sensitization to aero-allergens.) Boys got about 20% more moderate, vigorous, and MVPA than girls did, but participated almost equally in sport. Average daily MVPA, of which about 30% was vigorous, was about 45 minutes in boys and 35 minutes in girls. Sport participation was comparable between sexes.

A majority of children with asthma and rhinitis, especially boys, reported being currently medicated for it; but prevalence of symptoms remained high in these groups. (Table 2) 94% of asthmatic boys were on asthma medication and 46% had wheezed in the past year, compared to 78 and 68% of asthmatic girls. 66% of non-asthmatic boys with rhinitis but no asthma were medicated for rhinitis and 88% of them had nose/eye symptoms, compared with 61% and 84% of non-asthmatic girls with rhinitis. However, sport participation was comparable across allergy groups (70–80% of boys, 70–84% of girls).

Consistent with earlier work [29] we found that the study population of 1137 subjects was a few percentage points likelier to have had a nutritional intervention, to be female, to have highly educated parents, and to be urban than the whole 15-year followup (Table 1) which we previously compared with the populations recruited at birth. [29] Similar small differences separated lung-healthy controls from children with respiratory conditions (Table 2) but socioeconomic or anthropometric differences between the groups were small.

**Fig 1:**
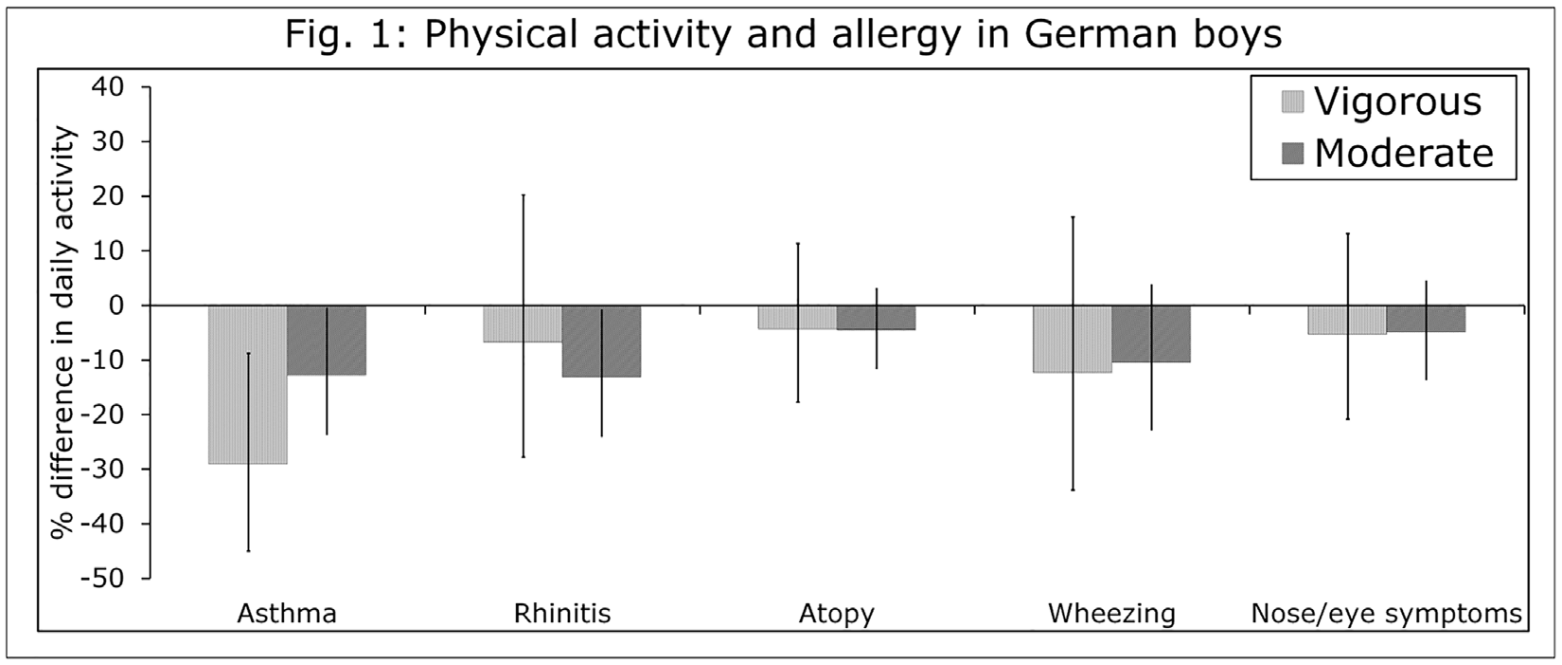
PA in adolescent German boys with asthma, rhinitis and atopy compared only to lung-healthy controls; boys with self-reported current wheezing or nose/eye symptoms compared to boys without these symptoms. All models corrected for age, height, nutritional intervention, study center (Munich or Wesel) and parental education. Error bars for 1.96 standard errors.

In boys but not girls, there was a clear trend towards less moderate and vigorous activity as sensitization increased from none, to atopy, to rhinitis, to asthma (Fig 1,Table 2) All associations were consistent whether we considered the mean, the geometric mean, or the 5th and 95th percentiles of PA (Table 2), but were of borderline significance. Control boys averaged 14.7 minutes VPA per day, which was 6% less in those with only atopy; 13% less with rhinitis but no asthma; and 24% less with asthma. This is similar to the observed levels of VPA in lung-healthy girls, who averaged 11.4 (8.8) minutes (Table 2). Moderate activity was also lower in allergic boys: controls got 31.8 minutes MPA per day, which was 2% less in those with only atopy, 10% less with rhinitis, and 7% less with asthma.

However, there were no significant associations between allergic phenotype and PA in girls whether we considered allergic conditions or symptoms. There was also no trend towards increasing or decreasing VPA or MPA across allergic conditions in girls. (Tables 2 and 3).

**Table 3:** Corrected Associations between PA and Allergic Phenotype. Models corrected for age, height, season of accelerometry, nutritional intervention, parental education and study center.

	Trait	Vigorous activity		Moderate activity		Any sport	
		% change	P	% change	P	Odds ratio	P
**Boys**	**Conditions**						
	Asthma^1^ (N = 53)	**-29.14**	**0.0076**	**-12.81**	**0.041**	0.63	0.18
	Rhinitis, no asthma^2^ (N = 50)	-6.83	0.59	**-13.14**	**0.037**	1.17	0.69
	Atopy, no rhinitis or asthma^3^ (N = 197)	-4.29	0.57	-4.53	0.23	0.91	0.66
	Lung-healthy ^4^ (N = 238)	(reference)	--	(reference)	--	(reference)	--
	**Symptoms (all subjects)**						
	Wheezing (N = 33)	-12.3	0.36	-10.48	0.14	**0.53**	**0.093**
	Nose/eye symptoms (N = 102)	-5.34	0.55	-4.97	0.29	1.24	0.41
**Girls**	**Conditions**						
	Asthma (N = 41)	23.02	0.14	9.67	0.24	2.02	0.14
	Rhinitis, no asthma (N = 40)	-1.26	0.93	-5.64	0.44	0.77	0.49
	Atopy, no rhinitis or asthma (N = 166)	-7.54	0.33	-5.96	0.16	1.46	0.11
	Lung-healthy (N = 352)	(reference)	--	(reference)	--	(reference)	--
	**Symptoms (all subjects)**						
	Wheezing (N = 40)	16.37	0.29	12.77	0.12	1.23	0.62
	Nose/eye symptoms (N = 93)	-5.32	0.57	-6.83	0.18	0.90	0.70

^1)^ Asthma: At age 15 the subject reported at least 2 of the following traits: doctor diagnosis of asthma at any time since age 3, asthma medication in past 12 months, wheezing in past 12 months, as in Jarvis et al (2012)

^2)^ Rhinitis: Current rhinitis or hayfever at age 15, but no asthma or asthma medicine

^3)^ Atopy: No asthma, no rhinitis, but one or more positive RAST (IgE ≥0.35) for aero-allergens (birch, mugwort, ambrosia, grass, rye, dogs, cats, dust mites *(Dermatophagoides pteronyssinus)* and indoor mold (*Cladosporium herbarum)*

^4)^ Lung-healthy: No asthma ever; no current rhinitis; no wheezing or nose/eye symptoms in past year; no current drugs for asthma or rhinitis; no RAST over 0.35 or positive bronchodilator response as defined in Miller et al (2005) and Flexeder et al (2015) [38]

**Bold text** if p≤ 0.10. P-values from generalized linear models treating VPA and MPA as lognormal, sport as binary (any vs. none.)

In corrected models, considering age, height, parental education, study center and nutritional intervention as covariates, boys with asthma got 29.1% less VPA (p<0.01, Table 2, Fig 1) than did control boys. They also got 12.8% less MPA, almost the same as boys with rhinitis (13.1%, both p = 0.04). Corrected estimates for the association between MPA, VPA and atopy in the absence of asthma and rhinitis remained negative but were very small (<5%) and non-significant (p>0.20.) No allergic symptom was significantly (p = 0.05) associated with any PA outcome in either sex (Table 2). No allergic diagnosis or symptom was associated with sport participation, and there was no consistent trend towards more or less sport with sensitization.

## Discussion

### Main Findings

In this large study of European adolescents we found that objectively-measured physical activity, particularly vigorous activity, was lower in adolescent boys with asthma and/or rhinitis, who nevertheless participated fully in sport; that symptoms did not appear to constrain PA; and that there were no effects in girls.

### Interpretation in Relation to Previous Work

Like others, [6]^,^[16] we found sex-specific associations between asthma, rhinitis and PA. However, while Groth et al. (2016) [6] and the current study found that asthma was associated with PA only in boys, Yiallouros et al. (2015)[16] found an effect only in girls, while Sugimoto et al[5] found that rhinitis was associated with lower PA only in boys. This heterogeneity may be explained by the known gender differences in prevalence, symptoms, and progression of asthma [39–42] and/or cultural differences in acceptability of sport [43, 44] especially for girls or children with asthma. [2] Furthermore, asthma diagnosis and treatment may be more available in our own population of Germans than in either the low-income Americans studied in Groth et al. (2016) [6] and Firrincieli et al. (2005)[13], or the Cypriots studied in Yiallouros et al. (2015)[16]. Lastly, our population was 2 years older than those in Groth et al. (2016)[6] and 7 years older than those in Yiallouros et al. (2015)[16] and age is known to impact on both activity pattern[45] and allergy development. [42]

We concur with the literature [2–4] that allergic phenotype, especially in boys, is often, but not always, associated with low PA; and find that neither respiratory symptoms[4] nor avoidance of sport[17] necessarily drive this difference. Direct comparison of effect sizes is problematic since some studies quantify PA as MVPA, some as VPA only, and some as sport participation; and at least in our cohort these measures of PA had different relationships with allergy. Furthermore many studies, including our own, are near the detection limit at p = 0.05 and thus are subject to effect inflation by publication bias. However, children with asthma or asthma symptoms typically got 30–50% less PA or sport in a recent review by Williams et al.[2] Asthma was associated with 30% less self-reported VPA and MPA in boys by Groth et al. (2016)[6] and 41% less accelerometric MVPA in girls by Yiallouros et al. (2015)[16] who like us found no effect of wheezing. Thus, our findings in boys are comparable[6] or at the lower end[2] of reported effects.

Intercomparison of studies is further hampered by the tendency to compare populations without correction for confounding, either by conditions such as overweight or by intercorrelations between allergic conditions. For example, Sugimoto et al. (2012) [5] found a 30% decrease in VPA among boys with rhinitis, but 35% of children with rhinitis also had asthma; and although rhinitis was not independently associated with VPA in our cohort, asthma was. Such comorbidity may have driven the association found by Sugimoto et al. (2012).

The lowered VPA in boys with asthma is consistent with the hypothesis that allergic symptoms or exercise-induced bronchoconstriction (EIB) may constrain PA. Wheezing was associated with 42% less accelerometric PA in Firrincieli et al. (2005)[13] and 30% less self-reported VPA in Groth et al. (2016.) [6] Despite a high percentage of medication use in our population, symptoms of asthma and rhinitis remained prevalent. Although we did not inquire whether it occurred specifically during exercise, wheezing is a common symptom of incompletely controlled asthma, typically occuring during VPA and/or cold weather.[18] In line with this observation, we found that in boys with asthma MPA was almost normal but VPA was much more significantly reduced, an observation also reported by Weisgerber et al. (2008) [46]. Likewise, Sugimoto et al. (2012)[5] found that in a population with a 35% prevalence of asthma MPA was almost normal, but VPA was obviously decreased.

Unlike others, we found that sport participation was not associated with any allergic diagnosis or symptom in either sex. However, we observed high rates of sport participation in general (over 2/3 of children) and previous research with this cohort [22, 47] found that neither female gender nor overweight was associated with sport. When combined with the fact that school sport is mandatory in Germany, it appears that sport is generally well accepted within this population and that barriers to participation are low. This was not the case in British populations studied by Williams et al. (2008), where asthmatic children avoid sport,[2] or are prevented from participating, [2] for fear of exacerbations.

Together, our data show less PA in boys with asthma and/or rhinitis even in the presence of comparable sport participation. Clinicians should fully control asthma and rhinitis in children in order to enable them to participate fully in VPA; and counsel these children and their parents of the possibility, demonstrated in this paper, of full participation in PA and sport. Activity-induced symptoms and consequent limitation of PA should be viewed as indicators to improve the treatment, ensure medication intake as prescribed, and thus eliminate the excuse for inactivity.

### Strengths and Limitations

Our subjects are a generally healthy, prosperous, and uniform group, recruited as follow-up from two birth cohorts and further selected for completion of interviews, a physical exam, wearing of an accelerometer, and meticulous keeping of diaries. Thus these adolescents are likely more compliant and thorough than their peers. They also live in a culture where PA and sport are common: sport participation levels are generally high, and we did not observe any tendency for lower participation in allergic adolescents or those with allergic parents (not shown.) This is known to not be the case elsewhere,[10] and relationships we find are thus necessarily population-specific.

However, since allergic symptoms and doctor diagnoses were reported by either participants or their parents ascertainment bias is a concern. Health-conscious individuals may recognize symptoms or remember diagnoses more accurately, or seek treatment more aggressively: however, relationships were stable in a sensitivity analysis limited to adolescents with negative bronchodilator response (not shown) suggesting that ascertainment bias did not drive results. Likewise, accelerometric data represent a snapshot of activity over a period of a week or less. While we made efforts to include only representative days during school time and to correct for seasonal effects, it is likely that some bias remains.

Lastly, sample size is a concern. Effect inflation by publication bias has been addressed above; to which we add that although our study population was large, it was mostly healthy. We sampled less than 100 adolescents with asthma and found an effect in only half of them (boys.) While no single outlier of either sex drove our results (not shown) and our model treated asthma and rhinitis as mutually exclusive (i.e. the model of rhinitis excluded adolescents with asthma) MPA and VPA were not. Because MPA and VPA tended to intercorrelate within subject (not shown) it is somewhat to be expected that a group of adolescents with low VPA (boys with asthma) also had low MPA.

### Implications for Future Research, Policy and Practice

Our research shows that at least some aspects of the relationship between PA and allergic phenotype are population-specific. Many previously found associations, particularly those between symptoms and PA and between sport and allergy, were not found in our population and thus may be modifiable. However, we confirm that asthma and rhinitis in boys are associated with low PA independent of each other and of reported symptoms, with effects most severe for asthma. We also find that even when allergic symptoms are present, allergic diagnoses do not necessarily preclude adolescents’ sport participation. Future research should further explore these links rather than taking them as proven.

Low PA by allergic children is in and of itself a health risk. Coaches, clinicians, and patients may need to collaborate to ensure adequate treatment of allergy and full participation in both sport and PA. To ensure equal access to the health benefits of PA, it may be necessary to target interventions or education to allergic children and/or their parents. PA performance should be specifically addressed by the treating physician, and limitations due to inadequate control of asthma and/or rhinitis should cause either improved treatment schemes and/or regular medication use by the child. Furthermore, counseling as to the need for PA should perhaps target the parents of allergic children, as well as the children themselves.

More generally, the risk of low PA was elevated in girls as compared to boys. Although asthmatic boys’ VPA was significantly lower than that of lung-healthy boys, it was almost the same as that of lung-healthy girls. This confirms previous research by us [23]and by others[44] [20] which shows that males are significantly more active than females. Since recommended PA levels are the same for both sexes, [48] this difference suggests that girls are particularly vulnerable to inactivity, and thus perhaps at greater risk for inactivity-related diseases. Future research and interventions should focus on increasing PA in girls.

## Conclusions

In adolescent boys, but not girls, asthma and rhinitis were independently associated with low PA. The association persisted in the absence of differential sport participation and did not appear to be explicable by activity-limiting allergic symptoms. Interventional data are needed to establish existence and direction of causation. Clinicians, parents, and designers of PA interventions should specifically address PA performance of boys with allergic diseases to ensure their full participation in sport.

## Supporting Information

S1 File Selection, Confounders and Statistics. (DOC)
