# Supplementary material for: Asthma and Rhinitis Are Associated with Less Objectively-Measured Moderate and Vigorous Physical Activity, but Similar Sport Participation, in Adolescent German Boys: GINIplus and LISAplus Cohorts
Source: PLoS One. 2016 Aug 25;11(8):e0161461. doi: 10.1371/journal.pone.0161461 (PMC4999273; doi:10.1371/journal.pone.0161461)
Supplement: S1 File — (DOC) [file pone.0161461.s001.doc]

## Selection:

Accelerometry participants were recruited from the entire 15-year followup of GINIplus and LISAplus that resided in the study centers Munich and Wesel. Attempts were made to contact subjects at age 15 by paper mail, electronic mail, and/or telephone, and at this time consent for accelerometry was requested. If consent was given, subjects received a postcard one week before the scheduled start time of accelerometry requesting consent to send the device. Those who confirmed consent by postcard were sent the device, and almost all returned the device after the scheduled week. For a flowchart see Figure 1 in Smith et al, [1].

Of the 7642 adolescents from Munich and Wesel who were initially recruited for GINIplus and LISAplus, 4306 were followed up at age 15. Of these, 2997 gave initial consent to accelerometry, 1682 completed accelerometry, and 1411 successfully passed data-quality checks. Of these, 1137 provided unambiguous data on respiratory comorbidities (see below, “Inclusion Criteria”) and thus were included in the current study. Dropout at all stages was very similar at all stages between GINIplus and LISAplus. For further details see Smith et al, [1].

For details on GINIplus recruitment see [2, 3] and the study’s website at ginistudie.de. For details on LISAplus recruitment see [4, 5] and the study’s website, [6].

All questions and protocols used in the current study were comparable between GINIplus and LISAplus. Height, weight, IgE RAST and bronchodilator response (BD) were objectively measured during the physical examination at 15 years. Information on other correlates (e.g. parental education) was obtained from standardized questionnaires from the initial survey (age 4 -6 months) and followups to 15 years.

## Confounders (sociodemographic and anthropometric):

**Age** and **height** were included in all models. Initial analyses suggested sex-specific results, so all presented models are stratified by sex.

**Body mass index** was quantified both as linear kg/m^2^ and categorically (underweight, normal, overweight, obese) based on age- and sex-specific 10^th^, 90^th^ and 97^th^ percentiles for BMI.[7] Overweight is often associated with asthma,[8] so we checked to see if this was the case in our population.

**Nutritional intervention:** The GINIplus cohort was initiated to investigate the relationship between hydrolysed baby formulas and subsequent allergy. Children with a family history of allergic disease were given one of four nutritional interventions in infancy (the intervention arm) while unselected children (controls) were given no formula. Because of nonrandom differences between the intervention and observation arms of GINIplus (see [1] for details) we corrected for nutritional intervention in all models. Since no intervention was used in LISAplus, all subjects from LISAplus were treated as controls.

**Study center:** All subjects were from either the urban environment of Munich, or the rural / suburban environment of Wesel. This difference may affect lung health, PA, or both; so we corrected for it in all models.

**Parental education:** Parental education was included in all models as a proxy for high socioeconomic status, measuring whether the higher-educated parent had entered college by the child’s fourth birthday. Roughly half of families achieved this cutoff.

**Season of accelerometry:** To avoid confounding by seasonal variation, all models were corrected for season of accelerometry as a four-level class variable. If the accelerometry began in March, April or May the season was “spring”; June, July or August were “summer”; September, October and November were “fall”; and December, January and February were “winter.”

# Statistical Methods:

# All calculations were done using SAS 9.2 or 9.3 (Cary, NC.)

Populations (Tables 1a and 1b) were compared using nonparametric tests: Kruskal-Wallis for multilevel categorical variables, Wilcoxon’s two-tailed rank-sum test for all others. To model corrected relationships, sex-stratified generalized linear models were used to model PA outcomes as statistical functions of respiratory diseases and conditions, corrected for age, height, study center, nutritional intervention, season of accelerometry (categorical) and parental education.

Each PA measure was modeled as statistical function of all confounders not of primary interest, as well as one respiratory condition or diagnosis at a time. MPA and VPA were log-transformed for normality before modeling. Inspection of histograms and q-q plots confirmed normality. “Any sport” was binary and modeled logistically.

**References**
